# Phenotypes of Sarcoidosis-Associated Pulmonary Hypertension—A Challenging Mystery

**DOI:** 10.3390/diagnostics13193132

**Published:** 2023-10-05

**Authors:** Aneta Kacprzak, Witold Tomkowski, Monika Szturmowicz

**Affiliations:** 1st Department of Lung Diseases, National Tuberculosis and Lung Diseases Institute, Plocka 26, 01-138 Warsaw, Poland

**Keywords:** sarcoidosis, pulmonary hypertension, phenotypes, treatment

## Abstract

Sarcoidosis has been a well-recognised risk factor for pulmonary hypertension (PH) for a long time, but still, the knowledge about this concatenation is incomplete. Sarcoidosis-associated PH (SAPH) is an uncommon but serious complication associated with increased morbidity and mortality among sarcoidosis patients. The real epidemiology of SAPH remains unknown, and its pathomechanisms are not fully explained. Sarcoidosis is a heterogeneous and dynamic condition, and SAPH pathogenesis is believed to be multifactorial. The main roles in SAPH development play: parenchymal lung disease with the destruction of pulmonary vessels, the extrinsic compression of pulmonary vessels by conglomerate masses, lymphadenopathy or fibrosing mediastinitis, pulmonary vasculopathy, LV dysfunction, and portal hypertension. Recently, it has been recommended to individually tailor SAPH management according to the predominant pathomechanism, i.e., SAPH phenotype. Unfortunately, SAPH phenotyping is not a straightforward process. First, there are gaps in our understanding of undergoing processes. Second, the assessment of such a pivotal element as pulmonary vasculature on a microscopic level is non-feasible in SAPH patients antemortem. Finally, SAPH is a dynamic condition, multiple phenotypes usually coexist, and patients can switch between phenotypes during the course of sarcoidosis. In this article, we summarise the basic knowledge of SAPH, describe SAPH phenotypes, and highlight some practical problems related to SAPH phenotyping.

## 1. Introduction

Pulmonary hypertension (PH) is a haemodynamic state of elevated blood pressure in the pulmonary artery shared by many conditions, and right heart catheterisation (RHC) remains the gold standard for its diagnosis [1]. For decades, 25 mmHg was the cut-off value for increased mean pulmonary arterial pressure (mPAP) at rest. Only recently, resting PH has been redefined as mPAP > 20 mmHg, and exercise PH was re-introduced with a new definition [1]. The haemodynamic categories of PH are presented in Table 1.

Another commonly used PH classification is the World Health Organisation’s (WHO) clinical classification. It consists of five main PH groups in which clinical conditions associated with PH are categorised on the basis of similar PH pathophysiological mechanisms, clinical presentation, haemodynamic characteristics, and management [1].

Sarcoidosis has been long recognised as a risk factor for PH. First, sarcoidosis-associated pulmonary hypertension (SAPH) was simply considered to be secondary to lung disease [2], but later observations showed that 30–40% of SAPH patients do not have lung fibrosis, and even 9–12% do not exhibit any lung parenchyma involvement [3,4,5,6,7,8,9]. Currently, it is acknowledged that sarcoidosis may lead to PH via multiple mechanisms, resulting in various forms of PH [1,10]. In PH clinical classification, SAPH belongs to WHO group 5, i.e., PH with unclear and/or multifactorial mechanisms [1]. The heterogeneity and complexity of SAPH are due to the heterogeneity of sarcoidosis itself. This systemic inflammatory disease of unknown cause may affect any organ, giving various clinical manifestations and consequences. Beginning with inflammation in the form of noncaseating granulomas, sarcoidosis may lead to fibrosis and/or failure of affected structures [11,12]. Both inflammation and fibrosis play a role in SPAH development. SAPH may result from parenchymal lung disease, the extrinsic compression of pulmonary vessels, pulmonary vasculopathy, LV dysfunction, or portal hypertension [1,10]. Sarcoidosis increases the risk of PH development by 3.2 times [13]. Additionally, sarcoidosis is associated with a three-fold increased risk of thromboembolic disease, which may implicate a higher risk for chronic thromboembolic pulmonary hypertension [14,15]. Also, the prevalence of obstructive sleep apnoea in sarcoidosis has been reported to be as high as 88% [16], and this could be another possible mechanism inducing SAPH. However, the impact of these comorbidities has not been studied enough.

Sarcoidosis is a rare disease, and its incidence and prevalence vary broadly depending on geographic region, race, age, and sex. The highest rates are reported in Northern Europe and in African American individuals, and the lowest in Asia. The estimated incidence in Europe ranges between 1 and 19 cases per 100,000 persons/year [12,17]. Resting SAPH prevalence, according to the previous PH definition, was estimated to be 6.4% in the general sarcoidosis population and 62.3% in the population with advanced pulmonary disease [18]. Pre-capillary PH dominated in SAPH [7,18,19], and 46% of pre-capillary SAPH was moderate to severe as defined using an mPAP > 35 mmHg [5]. An increase in the resting SAPH prevalence by around 60% may be anticipated following the new PH definition [20], and a proportion of sarcoidosis patients with exercise PH is yet to be determined [21].

It remains unknown why some patients develop PH and some do not, despite the same demographic and sarcoidosis-related characteristics [5]. The role of genetic susceptibility is still under research [22].

SAPH is an independent risk factor for mortality in sarcoidosis and carries up to a 10-fold increase in the risk of death [13,23,24,25,26,27,28]. An estimated 5-year transplant-free survival in SAPH is 40–62% [8,19,29,30]. The predictors of adverse outcomes in SAPH are WHO-functional class 4, right ventricular dysfunction, moderate or severe lung fibrosis, 6 min walking distance (6MWD) < 300 m, total lung diffusion capacity for carbon monoxide (TLco) < 35%pred, and a persistent high level of N-terminal brain natriuretic pro-peptide (NT-proBNP) level after 3–9 months of treatment with vasodilators [6,19,30].

There are no strong recommendations for SAPH treatment, as evidence from randomised clinical trials (RCT) is limited [1,10]. Given the diversity of mechanisms leading to SAPH, the most current guidelines advocate phenotyping, i.e., searching for the predominant mechanisms of SAPH in each patient and then applying the most appropriate treatment directed to the underlying condition [10]. Lung transplantation (LTx) should be considered if all other available modalities of treatment fail [1,10].

Unfortunately, SAPH phenotyping is not a straightforward process. It requires sophisticated diagnostic modalities, and the assessment of some elements, such as vascular remodelling, pulmonary angiitis, or microangiopathy, remains beyond the capability of non-invasive tools. Mathijssen et al. have shared the outcome of their attempt to assess the prevalence of different SAPH phenotypes in a single-centre cohort of 40 patients [7].

## 2. SAPH According to the Predominant Pathophysiological Mechanism

### 2.1. Parenchymal Lung Disease

Sarcoidosis affects the lungs and thoracic lymph nodes in more than 90% of patients [31]. In the majority of affected people, acute granulomatous inflammation is self-limiting and resolves spontaneously with time. In about one-third of the cases, the inflammation becomes chronic. Subsequently, about 20% of patients with persistent inflammation develop lung fibrosis [32]. Based on chest X-ray appearance, five stages of intrathoracic sarcoidosis are distinguished, as presented in Table 2 [33].

Pulmonary involvement increases the risk of PH almost five times compared to non-pulmonary sarcoidosis. In the population from the Nationwide Inpatient Sample 2016–2018, the prevalence of PH was 19% in cases with pulmonary sarcoidosis and 3.5% with non-pulmonary sarcoidosis; however, there was no information on pulmonary sarcoidosis staging [34]. In published research, 65–80% of sarcoidosis patients with pre-capillary PH confirmed by RHC had stage 4 disease, as assessed using either chest X-ray or HRCT [4,5,6,7,8]. In about half of these patients, PH was mild, i.e., mPAP ≤ 35 mmHg [4,5]. Surprisingly, no correlation could be found between mPAP or pulmonary vascular resistance (PVR) and spirometric or plethysmographic parameters, and patients with similar radiologic and pulmonary function characteristics behaved differently in terms of PH development [4,5,8,24,35].

The ablation, compression, and distortion of pulmonary vessels, together with hypoxic pulmonary vasoconstriction (HPV), are believed to be the first elements of the pre-capillary PH development pathway in the parenchymal phenotype of SAPH by analogy to other chronic lung diseases [36,37]. HPV is a compensatory mechanism to maintain ventilation/perfusion homeostasis by diverting blood flow from more seriously to less seriously affected lung segments. Depending on the magnitude of the pulmonary vascular bed loss and the magnitude of HPV, pulmonary artery pressure may increase, albeit usually mildly. In some yet not identified circumstances, the increased shear stress from high blood flow induces a response from pulmonary vessels that leads to their remodelling and irreversible structural changes, resulting in significant elevations of PVR and PAP. This interaction between increasing shear stress and pulmonary vasculature remodelling may continue in a vicious cycle [38,39,40,41,42]. Remodelling involves all levels of the pulmonary vasculature and all layers of the vessel walls [40,43,44,45]. ^18^F-fluorodeoxyglucose (^18^F-FDG) uptake was found on positron emission tomography (PET) in the pulmonary artery wall in 19% of 175 sarcoidosis patients, and these patients had higher PAP and PVR compared to patients without the uptake [46]. The meaning of this phenomenon is not clear, but one of the theories is that it reflects the inflammation caused by the shear stress and could be helpful in the early detection of developing PH [47].

The distinctive features of sarcoid inflammation and fibrosis, including a specific lymphangitic fashion of distribution, can be responsible for differences observed between SAPH and PH due to other interstitial lung diseases (ILDs). For example, the same degree of fibrosis is associated with higher PAP in sarcoidosis than in idiopathic pulmonary fibrosis (IPF) and other ILDs [48,49]. The predilection to bronchovascular bundles, septa, and subpleural regions may be associated with a larger direct impact on nearby pulmonary vessels [50].

A clear definition of a parenchymal SAPH phenotype is lacking. Mathijssen et al. adopted the recommendations regarding PH associated with chronic lung diseases from the latest World Symposium on PH to define the criteria of a parenchymal SAPH. These criteria include pre-capillary PH and moderate to severe pulmonary parenchymal disease; the latter was defined as stage 3 or 4 pulmonary sarcoidosis or severe obstructive (forced expiratory volume in 1 s (FEV_1_) ≤ 60%pred) or restrictive disease (forced vital capacity (FVC) ≤ 70%pred). They found the parenchymal phenotype in 73% of the patients from their SAPH cohort. Interestingly, not all stage 4 patients with pre-capillary PH were classified as the parenchymal phenotype despite the used definition; 17% of them were assigned to a compression phenotype, and one was diagnosed with chronic thromboembolic PH. Patients with stage 2 pulmonary sarcoidosis also fulfilled the criteria of the parenchymal phenotype. Additionally, one patient with stage 2 disease was categorised as having a parenchymal phenotype despite not fulfilling the criteria, but it was decided that this category was the best fit [7].

There are no evidence-based recommendations for the treatment of SAPH with predominant parenchymal causes. As for now, the treatment of underlying diseases and the symptomatic treatment of respiratory and/or heart failure, if needed, is a standard of care [1,10]. If SAPH is accompanied by active parenchymal inflammation, treatment with glucocorticoids and/or other anti-inflammatory medications is warranted. However, there is no solid proof of their effectiveness [10,31,51,52]. The use of immunosuppressive therapy in a SAPH setting is very common, it was applied in 26%-77% of SAPH patients from registries [8,19] and in up to 80% of retrospectively analysed cohorts [7], including SAPH with fibrotic pulmonary disease [53]. Reports on treatment outcomes are very limited and divergent [4,8]. Of note, haemodynamic improvement has been observed in some SAPH patients with stage 4 pulmonary disease, and reporting authors speculated the effect depended on a component of active inflammation [8]. There is clinical, histopathological, and metabolic evidence that the majority of patients with fibrotic pulmonary sarcoidosis have concomitant active granulomatous inflammation [54,55,56,57,58,59]. Up to 85% of patients with stage 4 pulmonary sarcoidosis showed active inflammation in lung parenchyma on ^18^F-FDG-PET [57,58,59]. Volumetric quantitative ^18^F-FDG-PET measurements showed a similar burden of active pulmonary inflammation in stage 4 as in stages 2–3, with the extent of the inflammation area corresponding to about 45% of lung volume [57]. Distinguishing the burnt-out disease from fibrotic pulmonary sarcoidosis with smouldering inflammation can be challenging when based only on conventional radiologic studies [60,61]. The usefulness of serum biomarkers in predicting the activity of sarcoidosis has been researched, but none appeared to be accurate enough [57,58,59,62,63,64,65].

Antifibrotic agents could potentially play a role in the treatment of SAPH due to lung fibrosis [10], but this has not been properly addressed yet. Only 12 patients with fibrotic pulmonary sarcoidosis, less than 2% of the studied population, were enrolled into the INBUILD study, RCT that investigated the efficacy of nintedanib in progressive fibrotic ILDs. Four were in the active treatment arm, and eight in the placebo arm. That did not allow for significant conclusions on the therapeutic effect of nintedanib in sarcoidosis [66,67,68]. No further patients with progressive fibrotic pulmonary sarcoidosis treated with nintedanib were identified in the systematic search through the literature until Dec 2020 [68]. Patients with sarcoidosis were not included in the RELIF study, which investigated pirfenidone in fibrotic ILDs [69]. The only RCT on pirfenidone in progressive fibrotic pulmonary sarcoidosis had to be stopped due to the COVID-19 pandemic after only 16 patients were enrolled. Fourteen patients, ten receiving pirfenidone and four receiving placebo, completed 18 months of the trial [70]. The rationale for the common use of anti-fibrotic drugs in pulmonary sarcoidosis is not strong. The progressive phenotype of fibrosis in sarcoidosis is not frequent compared to other fibrosing ILDs, it has been seen in about 15–50% of patients with fibrotic pulmonary sarcoidosis [28,71,72]. Only one patient fulfilling the criteria of progressing fibrotic pulmonary sarcoidosis was found among 167 patients with progressive fibrosing ILDs hospitalised in the years 2012 and 2021 in two hospitals in Osaka, Japan [73]. The usual interstitial pneumonia (UIP) pattern is very rare in fibrotic pulmonary sarcoidosis [28,55,68,74]. It is still unknown whether these rare cases of UIP pattern in sarcoidosis are a form of fibrotic sarcoidosis, constitute a separate condition called combined sarcoidosis and IPF (CSIPF), or are only a fortuitous overlap [74]. If fibroblastic foci and honeycombing are present in pulmonary sarcoidosis, they are less numerous [55,56]. Fibroblastic foci from lungs with end-stage sarcoidosis and lungs with IPF differ to some extent in gene and protein expression [75]. Reports on the relationship of the UIP pattern with progressive fibrotic pulmonary sarcoidosis are inconsistent, although the UIP pattern is associated with a worse prognosis [28,74]. Also, not much is known about the impact of progressive fibrotic pulmonary sarcoidosis on PH. In one report, PH was more prevalent in progressive (24%) compared to non-progressive (10%) fibrotic pulmonary sarcoidosis, but that difference was not statistically significant [28].

The use of pulmonary vasodilators in SAPH is still an open and controversial topic. Given the vascular remodelling processes appearing in SAPH on one hand, and the beneficial outcome from treatment with inhaled NO, inhaled treprostinil, and sildenafil shown in group 3 PH [76,77,78] on the other hand, some advantages from pulmonary vasodilator could be expected in the parenchymal phenotype of SAPH. The majority of reports on vasodilators in SAPH are retrospective case reports with inconsistent observations regarding the efficacy of the treatment [6,8,29,30,79,80,81,82,83,84,85,86]. Few prospective open-label studies were not conclusive either [87,88,89]. First, inhaled iloprost was assessed in the trial that enrolled 22 patients, but only 15 completed the 16-week therapy. Six patients showed haemodynamic improvement expressed as a decrease in PVR of ≥20%, and five also had a reduction in mPAP of ≥5 mmHg. Only three patients improved 6MWD by ≥30 m. A significant improvement in quality of life (QoL) was noted in some patients [87]. Next, ambrisentan was studied in a cohort of 21 patients, of which only 48% completed the 24-week therapy. No significant improvement was achieved in 6MWD, NT-proBNP, TLco, or QoL [88]. The last open-label trial was designed to study the efficacy of tadalafil, and 12 patients were enrolled, but only a small portion completed the 24-week period of therapy. No benefit was shown [89]. Only two RCTs dedicated to pulmonary vasodilators use in SAPH have been conducted so far, one with bosentan and the other with riociguat [53,90]. The trial with bosentan involved 35 patients, 23 assigned to the active treatment arm and 12 to the placebo arm, 50% with fibrotic lung disease, and lasted for 16 weeks. Bosentan improved haemodynamic parameters but had no effect on 6MWD [90]. The trial with riociguat enrolled 16 SAPH patients, randomised 1:1 to active treatment or placebo, 75% with lung fibrosis, and lasted 1 year. Riociguat improved 6MWD and was effective in preventing clinical worsening. Changes in pulmonary haemodynamics were not assessed [53]. Overall, published reports indicate that the use of pulmonary vasodilators in SAPH is safe and well tolerated [6,8,30,53,79,80,81,82,83,84,85,86,87,88,89,90]. The risk of blood oxygen saturation worsening is minimal, practically limited to the use of prostacyclin analogues administered either intravenously or subcutaneously, and even in those cases, it was only transient, resolving within 3–4 weeks [53,84,89,90]. The limitations of available retrospective and prospective studies include not only small samples and diversity of analysed end-points but also heterogeneity with respect to pulmonary sarcoidosis stages and the concomitant use of immunosuppressive medications, which could have had a significant impact on the findings. The results of three phase 2 studies are awaited: (i) SPHINX trial (NCT03942211), an RCT to assess efficacy and safety of oral selexipag in SAPH, (ii) SAPPHIRE trial (NCT03814317), an open-label study with inhaled treprostinil for stage 4 pulmonary sarcoidosis with pulmonary hypertension, and (iii) an open-label study to assess the safety and efficacy of increasing doses of pulsed, inhaled nitric oxide (NO) in subjects with PH associated with pulmonary fibrosis or sarcoidosis on long term oxygen therapy followed by long term extension study (NCT03727451). It is also noteworthy that attention has been paid to the potential anti-inflammatory and anti-fibrotic effects of vasodilators [91,92,93,94]. Inhaled treprostinil improved FVC %pred and lowered the risk of clinical worsening and exacerbation of ILDs underlying PH in the INCREASE study [92]. Treatment with PH-targeted medications prevented FVC decline in patients with SAPH evaluated for LTx [93]. On the other hand, a prospective 12-month phase 2 RCT found no evidence for the efficacy of bosentan as an anti-fibrotic treatment for steroid-resistant pulmonary sarcoidosis [94]. Change in FEV_1_ and FVC has been planned as a secondary end-point in the above-mentioned ongoing SAPPIRE trial with inhaled treprostinil for SAPH. Currently, there are no approved PH-targeted therapies for SAPH. Off-label uses of pulmonary vasodilators may be considered for symptomatic patients on a case-by-case basis [1,10]. They may be also useful in patients referred for LTx, potentially decreasing mortality while waiting on the list [93].

### 2.2. Extrinsic Compression of Pulmonary Vessels

The patency of pulmonary vessels, both arteries and veins, may be compromised by extrinsic compression caused by active sarcoidosis-based inflammation, fibrous lung parenchyma, lymph nodes, or fibrosing mediastinitis. The prevalence of mediastinal lymphadenopathy in sarcoidosis exceeds 90% [33]. The prevalence of fibrosing mediastinitis is unknown, but sarcoidosis may account for about 11% of fibrosing mediastinitis [95] and 42% of fibrosing mediastinitis causing PH [96]. The phenomenon of extrinsic vessel compression and its significance in SAPH development is poorly explored and understood [4,7,8,10,97,98,99]. The frequent compression of proximal pulmonary arteries, occasionally with luminal obliteration, has been revealed in the postmortem examination of the lungs of sarcoidosis patients [100]. Mathijseen et al. defined compression phenotype as a precapillary PH with the compression of central or segmental pulmonary arteries in imaging studies. They found this phenotype in 15% of their patients, with compression caused by either lung fibrosis, fibrosing mediastinitis, calcified lymph nodes, or active inflammatory masses. Among these six patients, five had stage 4 pulmonary sarcoidosis, and one had stage 1 disease. In two patients, including one with stage 1 pulmonary sarcoidosis, compressing structures were metabolically active on ^18^F-FDG-PET [7]. In a French cohort of 126 patients with severe pre-capillary SAPH, 4% had extrinsic compression of pulmonary arteries due to lymphadenopathy (two cases) or fibrosing mediastinitis (three cases). An increased uptake of ^18^F-FDG on PET was present in three patients: two with compressive lymph nodes and one with fibrosing mediastinitis [8]. In another cohort of 22 pre-capillary SAPH patients, the extrinsic compression of large pulmonary arteries by lymph nodes was recognised in 14% of the patients, all stage 4 disease [4]. In a meta-analysis performed on 17 SAPH patients who underwent pulmonary angioplasty for focal stenosis or external compression of the pulmonary vessels, the most frequently affected were the right pulmonary artery and its branches, and the least frequently affected were the pulmonary veins. Usually, multiple bilateral stenosis was present [97]. Extrinsic pulmonary vessel compression may appear in any stage of pulmonary sarcoidosis [98] and may be detected on contrast-enhanced CT, HRCT, or pulmonary angiography [4,7,8,97,98].

The assumption that the compressive phenotype of SAPH is confined to pre-capillary PH seems controversial and may be responsible for the underestimation of the prevalence of this phenotype. In a cohort of 27 patients with fibrosing mediastinitis and PH, 18% had post-capillary PH without concomitant cardiac disease. Sarcoidosis patients accounted for 48% of the whole group. In 52% of the patients, severe compression of pulmonary veins was present, the most probable cause of elevated pulmonary arterial wedge pressure (PAWP) [96].

Immunosuppressive treatment was reported to be beneficial in some patients with extrinsic pulmonary vessel compression but ineffective in others [7,8,96,98,99]. Such treatment may be effective in acute granulomatous inflammation but has no effect when fibrosis develops [7,8,96,98,99]. The experience with ^18^F-FDG-PET as a tool predicting response to the treatment is limited and shows not satisfying accuracy [7,8,96]. Pulmonary vasodilators have been tried in a few cases, but the small sample did not allow for any conclusions [96]. Balloon angioplasty with or without stenting seems to give significant haemodynamic and functional improvement [97,98]; however, currently, it is an off-label treatment option to be considered only in carefully selected patients with focal and critical vessel stenosis [10,97]. It is recommended to evaluate for pulmonary artery stenosis or mediastinal compression on chest imaging in all SAPH patients [10].

### 2.3. Pulmonary Angiitis and Microangiopathy

Mathijssen et al. defined a vasculopathy phenotype as a pre-capillary PH with PVR > 3 Wu, no or mild pulmonary disease, and excluded other causes of PH. Based on that, they classified only 1 patient of the group of 40 as a suspected vasculopathy phenotype [7]. Yet, the lung pathological evaluation proves that intrinsic vascular involvement at all levels of pulmonary vasculature is very common in sarcoidosis. It appears in two forms: sarcoidosis-specific granulomatous angiitis and non-specific microangiopathy [4,100,101,102]. Granulomatous pulmonary angiitis means the presence of granulomas within walls of blood vessels with the destruction of lamina elastica and was found in 100% of sarcoidosis cases in specimens from autopsy series [100], in 69% of specimens from open lung biopsies [102], and in 41% of transbronchial biopsies [101]. It was also found in 80% of explanted SAPH lungs [4]. Both granulomas and healed lesions of various stages were observed, often coexisting. Granulomatous vascular involvement correlated positively with the extent of granulomas in lung parenchyma, but there was no correlation between the occurrence of granulomatous angiitis and radiographic pulmonary sarcoidosis stages [100,101]. Interestingly, venous involvement predominated and accounted for up to 90% of cases [4,100,101,102]. Granulomas were found in vessels of all calibres, but 75% of the involved veins and 54% of arteries were less than 100 µm in diameter [101]. Microangiopathy refers to the alterations of endothelial and basement membrane of precapillary, capillary, and postcapillary pulmonary vessels and was found in 35% of transbronchial biopsy specimens from patients with sarcoidosis [101] and 100% of explanted lungs of five patients with SAPH [4]. Both angiitis and microangiopathy caused vessel occlusion and destruction [4,45,50,100,101,102,103,104]. Plexiform lesions were not typically seen in sarcoidosis-associated vasculopathy [4,100,101]; however, venous involvement with vessel obliteration and subsequent capillary congestion may resemble pulmonary veno-occlusive disease (PVOD) [4,105,106]. The assumption that pulmonary vasculopathy is to be expected only in cases with mild pulmonary disease and severe PH, in the absence of other possible causes, is debatable in light of the facts presented above. Most probably, it contributes significantly to the development of SAPH believed to be of predominantly different phenotypes, and it may be responsible for severe or out of proportion to perceptible factors PH. Also, given the vast venous involvement, a post-capillary PH could be expected. A negative correlation between mPAP or PVR and TLco was found in SAPH, and septal lines and ground glass opacifications appeared more often on the lung HRCT of SAPH patients compared to sarcoidosis patients without PH [4,7]. These clinical and radiologic features are known to occur commonly in PH with overt involvement of pulmonary veins [1]. Pulmonary vasculopathy is potentially the most underappreciated mechanism of SAPH, as it is difficult to establish the diagnosis and to assess the extent of vascular involvement antemortem.

Immunosuppressive agents are suggested as the first-line treatment when sarcoidosis vasculopathy is suspected, with the option of the pulmonary vasodilators used in some patients [10]. In patients with venous involvement resembling PVOD, pulmonary vasodilators should be used carefully in order to avoid worsening pulmonary congestion [1,10].

### 2.4. LV Dysfunction

It is believed that post-capillary SAPH results from cardiac involvement in the course of sarcoidosis that causes LV function impairment. This hypothetical cause-and-effect relationship has not been validated practically, though no studies addressing this topic can be found. Cardiac involvement has been observed in up to 27% of Caucasian and African American sarcoidosis patients and even up to 80% of Japanese patients; in the majority of them, it was clinically silent [107,108,109]. Post-capillary PH accounted for 7–28% of SAPH cases in reported groups [5,7,19]. In the group reported by Mathijssen et al., none of the patients with post-capillary SAPH had cardiac sarcoidosis, and all had preserved systolic LV function [7]. In another study, 65% of post-capillary SAPH patients had normal LV ejection fraction, data on cardiac involvement were not reported. In that study, patients with post-capillary SAPH and impaired LV function were clinically indistinguishable from patients without LV disease but had better clinical outcomes [24]. It is also interesting that no pulmonary hypertension was observed in a French cohort of cardiac sarcoidosis patients despite systolic or diastolic LV dysfunction in many of them [109]. These data may undermine the theory of such a straightforward cause-and-effect relationship between post-capillary SAPH and cardiac sarcoidosis. An alternative explanation could include the known vast intrinsic and extrinsic involvement of pulmonary veins of all calibre in sarcoidosis [4,97,100,101,102]. In the cohort of sarcoidosis patients listed for LTx, the PAWP was significantly higher in the group with PH compared to patients with no PH [35]. Unfortunately, patients with post-capillary PH are often excluded from studies on SAPH [5,6,8], and reports on cardiac sarcoidosis do notexplore this intriguing plot.

In post-capillary SAPH due to cardiac sarcoidosis, glucocorticoids and other immunosuppressive agents are recommended. In addition, the standard treatment for cardiac failure should be applied as appropriate [1,10,51]. This SAPH phenotype requires more attention in future studies.

### 2.5. Portal Hypertension

The liver involvement can be found in 50–75% of sarcoidosis patients, but symptomatic disease occurs in only 5–15% [110,111]. Portal hypertension (PoH) develops in less than 1% of sarcoidosis patients [111] and in up to 33% of patients with proven hepatic sarcoidosis [110]. PoH can be caused by biliary cirrhosis or the compression of the portal vein by involved lymph nodes or parenchymal granulomas, as well as sarcoid hepatic vasculitis [110,111]. It is believed that 2–6% of patients with portal hypertension of various aetiology develop pulmonary arterial hypertension, also called portopulmonary hypertension (PoPH) [1]. The overall occurrence of PoPH in sarcoidosis seems very rare, but it should not be forgotten in a differential workup of SAPH.

Glucocorticoids and other immunosuppressive agents are recommended for PH caused by sarcoid liver disease [10]. Pulmonary vasodilators should also be considered [1].

## 3. Conclusions

The true prevalence of SAPH and the prevalence of its phenotypes based on predominant cause remain unknown. In the era of precise and personalised medicine, such phenotyping is recommended to lead the management. However, SAPH phenotyping is not a straightforward process. Apart from lacking criteria and gaps in knowledge, multiple phenotypes usually coexist and patients can switch between phenotypes during the course of sarcoidosis. Sarcoid pulmonary vasculopathy is probably the most under-recognised pathophysiological factor of SAPH development, and post-capillary phenotype seems the least studied. Whether genotypes play a role in SAPH development or SAPH phenotype needs further studies [22,112,113]. The complexity and distinctive features of SAPH do not allow for the direct translation and adoption of the findings from research on PH belonging to other WHO groups.

A transthoracic echocardiogram (TTE) should be performed as an initial assessment in all sarcoidosis patients presenting features indicative of PH [10,114,115]. RHC is recommended in patients with high TTE probability of PH, in patients with intermediate probability of PH and FVC ≥ 50%pred, and in patients being considered for LTx [10]. Given the current understanding of SAPH and available therapeutical options, the pivotal step in its management is the recognition of an ongoing inflammation warranting immunosuppressive treatment. ^18^F-FDG-PET seems to be the most helpful tool to achieve this goal; however, it has not been validated and standardised in this indication. There is a large need for well-planned research projects to properly characterise the epidemiology and phenotypes of SAPH and to establish an evidence-based management for this condition. It is also very important to take sarcoidosis into the differential workup of PH, remembering that it may cause PH without visible involvement of the lungs or thoracic lymph nodes.

## Figures and Tables

**Table 1:** Haemodynamic categories of pulmonary hypertension [1].

PH Category	Definition
pre-capillary PH	mPAP > 20 mmHg, PAWP ≤ 15 mmHg, PVR > 2 WU
isolated post-capillary PH	mPAP > 20 mmHg, PAWP > 15 mmHg, PVR ≤ 2 WU
combined pre- and post-capillary PH	mPAP > 20 mmHg, PAWP > 15 mmHg, PVR > 2 WU
unclassified PH	mPAP > 20 mmHg, PAWP ≤ 15 mmHg, PVR ≤ 2 WU
exercise PH	mPAP/CO slope between rest and exercise > 3 mmHg/L/min

PH—pulmonary hypertension, mPAP—mean pulmonary arterial pressure, PAWP—pulmonary arterial wedge pressure, PVR—pulmonary vascular resistance, WU—Wood units, CO—cardiac output.

**Table 2:** Stages of intrathoracic sarcoidosis [33].

Stage	Features
0	no visible intrathoracic findings
1	enlarged hilar lymph nodes only
2	enlarged lymph nodes and parenchymal infiltrates
3	diffuse parenchymal infiltrates without lymphadenopathy
4	lung fibrosis

## Data Availability

Not applicable.
